# Survival of a patient with an acute traumatic subdural hematoma and high-grade liver injury with associated IVC injury without surgical intervention

**DOI:** 10.1016/j.tcr.2025.101194

**Published:** 2025-05-26

**Authors:** Patrick T. Lee, Elliot Jessie, David T. Efron

**Affiliations:** a University of Maryland School of Medicine, R. Adams Cowley Shock Trauma Center, 22 South Greene Street, Baltimore, MD 21202, USA; b Walter Reed Army Medical Center, 4494 Palmer Road N, Bethesda, MD 20814, USA

## Abstract

Blunt trauma can often result in multi-organ injury. Traumatic brain injury with significant intracranial hemorrhage is usually addressed with surgical intervention to prevent further injury, particularly in the presence of midline shift. Concurrent liver injury and hemorrhage can present challenges in management. Here, we discuss the case of a 46-year-old male who presented after a motor vehicle crash and was found to have a subdural hematoma (SDH) with midline shift and herniation, which was determined to be non-survivable by neurosurgery. On cross-sectional imaging, the patient was additionally found to have a Grade V liver injury with extension into the inferior vena cava. No surgical interventions were performed for the intracranial hemorrhage or intra-abdominal injuries. The patient did not succumb to these injuries, but had a decrease in the size of the intracranial hemorrhage, and eventually regained consciousness during the hospital course, surviving to discharge.

## Introduction

Significant blunt polytrauma can present challenging management decisions for trauma surgeons. Individual cranial, chest, and abdominal injuries each have considerable morbidity and mortality, but in combination, the risks are increased. A subdural hematoma/hemorrhage (SDH) is a traumatic bleed that can result in significant morbidity and mortality. Surgical intervention is indicated depending on size, timing after trauma, and patient condition. SDHs can exert a mass effect on the brain, causing herniation of the brain stem resulting in eventual mortality [1]. Early signs of herniation include fixed and dilated pupils due to compression of cranial nerve three [2]. When head injuries occur with concurrent chest or abdominal trauma, the management is challenging as visceral injuries may take priority [3]. At times, simultaneous procedures to control abdominal or thoracic bleeding and temporizing methods to decompress the blood in the head until a formal craniotomy can be done are required. Significant abdominal trauma resulting in severe liver and inferior vena cava (IVC) injuries can be fatal if not intervened upon in a timely fashion in hemodynamically unstable patients. Retrohepatic IVC injuries represent a significant surgical challenge and have a high mortality rate despite interventions. In hemodynamically stable patients with high-grade liver injuries and IVC injuries, non-operative management has been described [[4], [5], [6], [7]]. Likewise, there are numerous reports of spontaneous SDH resolution without intervention, despite rapid surgical intervention being the standard of care for SDH with midline shift [8,9]. Here, we discuss a patient who presented to the trauma center following a motor vehicle crash. The patient had minimal signs of external trauma but was found to have a Grade V liver injury with extension to the IVC and a SDH with midline shift. Despite no initial intervention, the patient survived and was eventually discharged from the hospital.

## Case Report

We report the case of a 46-year-old patient who presented to the trauma bay as an unknown “Doe” after a motor vehicle crash traveling at an unknown rate of speed. Per the Emergency Medical Service (EMS) report, the patient may have hit several cars before coming to a stop, and airbags were deployed. Bystanders also reported to EMS that the patient was pulled from the vehicle and assaulted. Upon EMS arrival, the patient was unconscious, but arousal improved, and the Glasgow Coma Scale (GCS) score was reported to be nine before transport. The GCS declined while in transit to the hospital, and EMS began bag-mask ventilation to maintain oxygenation. On the primary survey, the patient was not protecting their airway, had a GCS of three, and was thus promptly intubated. The initial blood pressure was 90 systolic by palpation, with a heart rate of 131. The patient was given a unit of whole blood with improvement of systolic pressure to the 160 s. Other vitals were a temperature of 36.6C, respiratory rate of 21, and SpO2 of 97 %. The remainder of the trauma survey revealed a laceration to the lip and bilateral non-reactive pupils. Focused assessment sonography in trauma (FAST) exam revealed a small amount of fluid in the pelvis. Labs were notable for a hemoglobin of 11.8, arterial blood gas of 7.12/60/125/19/−10.8, and lactate of 7.8. A toxicology screen was positive for benzodiazepines, cocaine, and fentanyl.

**Fig. 1:**
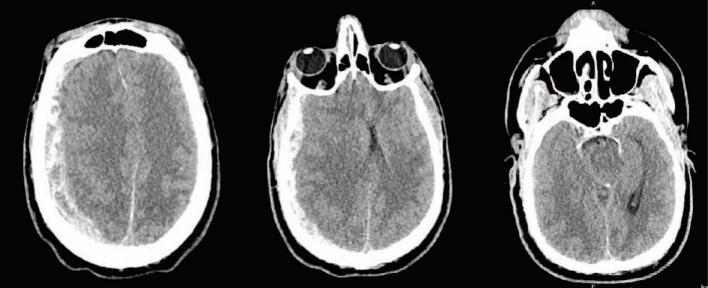
Admission CT Head. CT Head on admission demonstrated a 1.3 cm right SDH with 1.2 cm of midline shift and evidence of transtentorial herniation.

**Fig. 2:**
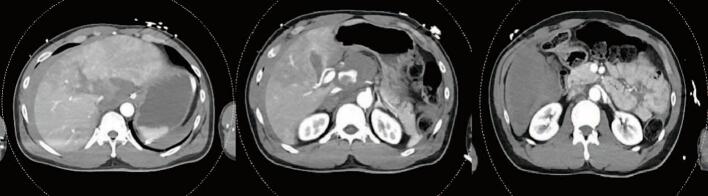
Admission CT Abdomen/Pelvis CT Abdomen/Pelvis showing a AAST Grade V liver injury with extension to the IVC and subhepatic hematoma. Additional bleeding from a branch of the right hepatic artery was seen in the right hepatic lobe.

With the improvement in blood pressure after transfusion, The patient was taken for a computed tomography (CT) scan of the head, cervical spine, chest, abdomen, and pelvis. They were found to have a 1.3 cm right SDH with a 1.2 cm leftward shift and transtentorial herniation (Fig. 1). Despite minimal evidence of external trauma, the patient was found to have an American Association for the Surgery of Trauma (AAST) Grade V liver laceration extending along the right and middle hepatic veins to the IVC, active arterial hemorrhage at the inferior margin of the liver and near the hilum - potentially from the hepatic artery, a right adrenal hematoma, and a large volume hemoperitoneum (Fig. 2). Additional injuries included multiple rib fractures, a scapular body fracture, traumatic pneumatoceles, and a right C7 transverse foramen fracture. After the CT scan was completed, the paralytic was reversed, and the patient was evaluated by neurosurgery. Repeat FAST exam now showed increased fluid in the abdomen. Neurosurgery evaluation determined that the head injury was non-survivable as the patient had bilateral blown pupils and absent corneal, cough, and gag reflexes. They were given 3 % saline followed by 1 g/kg of mannitol with no improvement. After a discussion between the trauma and neurosurgery teams, neurosurgical intervention was not recommended. Because the head injury was deemed to be non-survivable, no operative intervention for the liver laceration and abdominal bleeding was pursued. The patient was admitted to the intensive care unit (ICU) while they were identified and the family was located. Before going to the ICU, the patient was started on norepinephrine to maintain blood pressure.

**Fig. 3:**
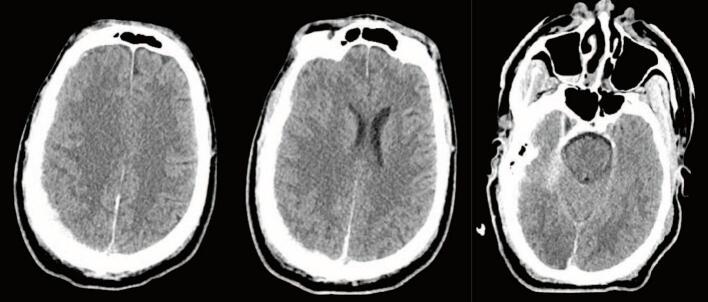
CT Head on Hospital Day 2 CT Head on HD2 showing decreased size of SDH now 6 mm and reduced midline shift of 6 mm.

**Fig. 4:**
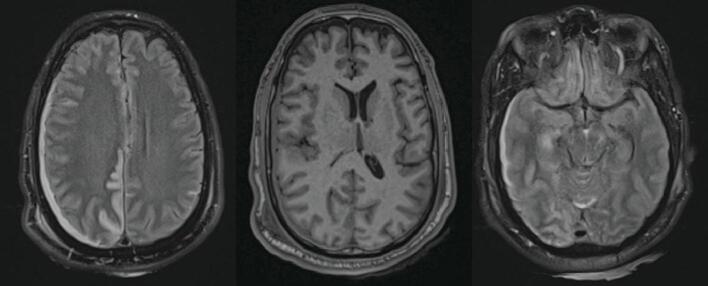
MRI Head on Hospital Day 2 T2-weighted MRI Head on HD2 showing multifocal acute infarcts secondary to herniation, 6 mm midline shift and stable transtentorial herniation.

The following morning on hospital day (HD) 2, while in the ICU, the patient was noted to have reactive pupils and brainstem reflexes; no intervention was recommended at that time. They underwent a repeat head CT, which demonstrated redistributed and decreased hemorrhage with decreased midline shift now at 0.6 cm and small subarachnoid hemorrhages (SAH) (Fig. 3). GCS at this time was still 3 T. Repeat abdominal imaging demonstrated no change in the Grade V liver injury, no active extravasation, and an unchanged hematoma adjacent to the IVC/hepatic vein confluence. A large right pneumothorax had also developed, for which a 14 Fr pigtail catheter was placed. The patient underwent an MR/MRA Brain, which demonstrated multifocal acute infarcts of the cerebral hemispheres, basal ganglia, rostral midbrain, and anterior cerebellum. The SDH and SAH were stable with 0.6 cm of midline shift (Fig. 4). Given the poor exam, an external ventricular device (EVD) was placed on HD 3; opening pressure was noted to be >20 cm H_2_0. Intracranial pressures (ICP) were treated with hypertonic saline. The patient became hypertensive, and blood pressure control was achieved with a nicardipine drip. On HD 4, they were noted to have seizure-like activity in the upper extremity and were given benzodiazepines. There was no evidence of seizure on an electroencephalogram, and GCS improved to 7 (M5V1E1). The patient was treated with bromocriptine, propranolol, and amantadine for paroxysmal sympathetic hyperactivity.

**Fig. 5:**
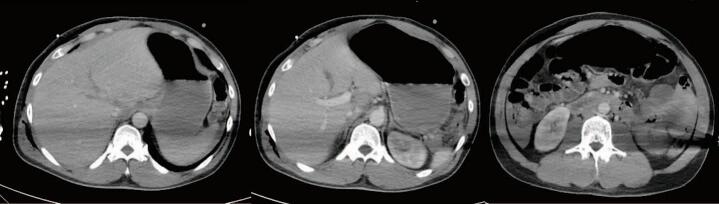
CT Abdomen Pelvis on Hospital Day 10 CT Abdomen and Pelvis on HD 10 demonstrated stellate liver laceration with healing and decreased hemoperitoneum.

**Fig. 6:**
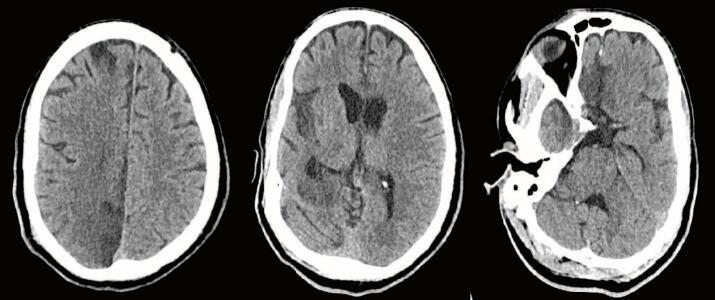
CT Head two months after discharge CT Head two months after injury showing resolution of the SDH with evolving encephalomalacia of right frontal and occipital lobes.

**Fig. 7:**
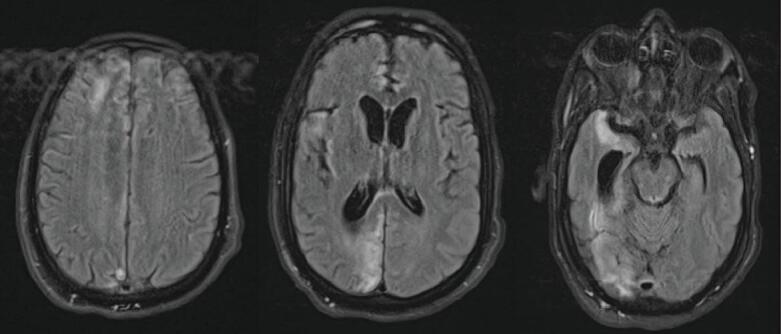
MRI Head two months after discharge T2-weighted MRI Head showing evolving multifocal encephalomalacia with right hemisphere volume loss and enlargement of right lateral ventricle.

The patient underwent tracheostomy and percutaneous gastrostomy tube placement, and a right 28Fr chest tube was placed for worsening subcutaneous emphysema and non-resolving pneumothorax on HD 6 (removed HD 10). The EVD was removed on HD 6. By HD 10, a repeat CT scan demonstrated the large liver laceration with some healing and decreased hemoperitoneum without evidence of a biloma or other fluid collections (Fig. 5). The patient was tolerating tube feeds. The GCS improved to 11 (M6V1E4) and intermittently followed commands, right> left. The patient was discharged to vent-weaning rehab on HD25 and was seen in the Neurosurgery clinic a month after discharge. The patient was noted to have a GCS of 15 and responded appropriately to questions, albeit with slowed speech, and only had some numbness in the left lower extremity. CT scan showed resolution of the SDH and evolving areas of encephalomalacia of the right frontal and occipital lobes (Fig. 6). MRI approximately two months after discharge showed evolving encephalomalacia and right hemispheric volume loss with ex vacuo enlargement of the right lateral ventricle (Fig. 7). Unfortunately, the patient was not seen again in the trauma clinic.

## Discussion

This patient presented with injuries, including a SDH with midline shift and herniation, a high-grade liver injury, and an IVC injury. Each injury has variable rates of morbidity and mortality, but together, they likely have a high mortality. Despite the high mortality risk of our patient, the patient survived without surgical intervention. The progression of acute SDH is often interrupted due to swift surgical intervention. Thus, the overall rate of spontaneous resolution is often unknown. Acute SDH with midline shift, diminished GCS, and evidence of impending herniation, if not treated rapidly, can be fatal. Medical treatment with hypertonic saline or mannitol can temporize a patient, but management includes burr holes, EVD placement for ICP monitoring and drainage, and a definitive decompressive craniotomy or craniectomy (1–3). Clinical guidelines proposed by Brain Trauma Foundation, Bullock and colleagues, and others put forth the following as indications for surgical intervention including loss of 2 points on the GCS scale, abnormal pupil examination, increased ICP > 20 mmHg, SDH thickness > 10 mm, and midline shift >5 mm [1,10]. Midline shift is associated with increased mortality; thus, surgical intervention is emergent [11,12]. Despite early interventions as described, there are numerous reports of spontaneous SDH resolution dating to the 1980s [[13], [14], [15]]. We additionally reviewed several case reports of spontaneous resolution of SDH in response to traumatic injury and a non-traumatic SDH [8,9,[16], [17], [18], [19], [20], [21], [22]].

In contrast to our patient, a number of the patients in other reports of spontaneous resolution had higher GCS scores on presentation or demonstrated improvement in their level of alertness without intervention. Those presenting with GCS <6 are often taken for surgical decompression. Fujimoto and colleagues were able to observe patients because surgery was not considered due to advanced age, poor prognosis, or family wishes. 32 % of these patients demonstrated a spontaneous resolution [23]. Several hypotheses have been proposed regarding the mechanism of spontaneous resolution. One proposed mechanism is the dilution and washout of the SDH by cerebrospinal fluid through a torn arachnoid membrane [13,14,21,24,25]. An alternative mechanism presented is the redistribution of hematoma to other spaces due to increased ICP. Increased subarachnoid space due to atrophy presents favorable conditions for the redistribution of blood [8,18]. Additionally, the evaluation of many of these spontaneous SDH resolutions sought to discover characteristics of the SDH that may predict spontaneous resolution. Several studies have evaluated a “low-density band” seen on CT scans, which represents CSF dilution of the SDH and the presence of coagulopathy [9,23]. Brooke and colleagues evaluated 154 patients with nonoperative SDH and did not find those factors consistent in patients with spontaneously resolved SDH [25]. Further work into the mechanism of spontaneous resolution and predictive factors remains to be done.

Patients presenting with abdominal injury or concern for hepatic injury will often undergo a CT scan to characterize the extent of the injury. Management consists of rewarming, fluid resuscitation or blood transfusion, and early intervention where warranted. Hepatic injuries are graded on a scale established by the AAST or World Society of Emergency Surgery (WSES) [26,27]. The higher the grade of injury, the higher the risk of morbidity and mortality, with Grade IV injuries having a mortality of 46 % and Grade V injuries having a mortality of 70–80 % [4,28]. The management of hepatic injuries has significantly changed over the last 50 years as treatment has included more non-operative management across all grades [4,[29], [30], [31], [32]]. Patients who are candidates for non-operative management include those who are hemodynamically stable on presentation, non-peritonitic, and have no other indication for surgical intervention. Hypotensive patients who respond to blood transfusions may be candidates for non-operative management [33]. Hypotensive patients in shock or those who present with peritonitis will often progress to the operating room for urgent surgical intervention, which could include hepatic packing, embolization, or hepatic resection, each of which has associated complications. At one point in history, traumatic hepatic resection had an estimated mortality of approximately 70 % [34].

Successful non-operative management in high-grade (Grade IV and Grade V) liver injuries has been demonstrated in multiple studies [5,[35], [36], [37]]. In many of these studies, the success rate was >90 %. Predictors of non-operative failure include systolic blood pressure of 100 mmHg or less and the presence of other abdominal injuries. Failure of non-operative management is often due to ongoing bleeding, which may require interventions [5,38]. According to the Eastern Association for the Surgery of Trauma (EAST) practice guidelines, angiography with embolization can be the first approach for bleeding [39]. Additional complications that may occur after non-operative management can include bile leaks, biloma, or hepatic necrosis. Kozar and colleagues estimated complication rates of 21 % in Grade IV liver injuries and up to 63 % in Grade V liver injuries requiring additional interventions such as embolization, endoscopic retrograde cholangiopancreatography (ERCP), or Interventional Radiology drainage [35]. Regardless, a majority of these patients were able to be successfully treated without surgical exploration.

High-grade liver injuries can be associated with significant bleeding from hepatic parenchyma, but bleeding from hepatic veins or the IVC is also possible. Although rare, IVC injuries with significant hemorrhage have mortality rates between 30 and 70 % [6,40,41]. The IVC is divided anatomically into the infrarenal, pararenal, suprarenal, retrohepatic, or suprahepatic. The infrarenal IVC is most commonly injured (39 %), followed by retrohepatic (19 %), suprarenal (18 %), pararenal (17 %), and suprahepatic (7 %). Depending on the location, mortality is due to exsanguination or cardiac tamponade [6]. IVC injuries are managed based on the location and severity of the injury, with important factors being hemodynamics on the presentation and location of the injury. Non-operative management can be pursued in hemodynamically stable patients in a monitored ICU before stepdown [37,39]. Numerous case reports of traumatic IVC injuries that were managed non-operatively demonstrate that these cases can be managed on an individual basis [6,7,42]. Endovascular therapies for venous injuries are not well studied, and no current devices exist for such injuries, but endovascular approaches have been undertaken in some cases utilizing an endograft in the retrohepatic IVC [43].

In hemodynamically unstable patients with actively bleeding lesions and other intra-abdominal injuries, operative intervention is required. Operative interventions to address significant liver bleeding include packing for compression and tamponade, suture ligation of bleeding vessels, electrocautery, the Pringle maneuver, and completed vascular isolation of the IVC. If significant bleeding occurs despite ongoing compression and a Pringle maneuver, an IVC injury should be suspected. Depending on the location, the intervention could require suture repair, or if the injury is destructive, ligation of the IVC might be necessary. Retrohepatic IVC injuries represent a challenging vascular injury that can occur with simultaneous liver injury. These injuries can have mortality upwards of 50–100 % even with operative intervention, with death occurring secondary to hemorrhage [44]. To gain access to the retrohepatic IVC, complete mobilization of the liver must occur, which takes time, and releasing the liver attachments contributing to tamponade can potentially lead to further hemorrhage. Hepatic packing or hepatic resection could be utilized to gain access or to control bleeding. Other interventions that can be done include balloon tamponade, atriocaval shunting, or veno-venous ECMO to obtain hemostatic control and allow vascular repair to occur [43,44]. As previously mentioned, these efforts take time, but if successful and with adequate resuscitation, these interventions can potententially result in a reduction in intra-abdominal bleeding and allow for repair of the injury.

## Conclusion

Significant intracranial hemorrhage with midline shift, high-grade liver, and IVC injuries are each individually associated with high rates of morbidity and mortality. In this case, we present a patient who presented with these injuries and survived without surgical interventions.

## CRediT authorship contribution statement

**Patrick T. Lee:** Writing – original draft. **Elliot Jessie:** Writing – review & editing. **David T. Efron:** Writing – review & editing.

## Declaration of competing interest

The authors declare that they have no known competing financial interests or personal relationships that could have appeared to influence the work reported in this paper.
